# Procollagen-Lysine, 2-Oxoglutarate 5-Dioxygenase Family: Novel Prognostic Biomarkers and Tumor Microenvironment Regulators for Lower-Grade Glioma

**DOI:** 10.3389/fncel.2022.838548

**Published:** 2022-02-18

**Authors:** Siming Gong, Changwu Wu, Franziska Köhler, Jürgen Meixensberger, Nikolas Schopow, Sonja Kallendrusch

**Affiliations:** ^1^Institute of Anatomy, University of Leipzig, Leipzig, Germany; ^2^Department of Neurosurgery, University Hospital Leipzig, Leipzig, Germany; ^3^Department of Orthopedics, Trauma and Plastic Surgery, Sarcoma Center, University Hospital Leipzig, Leipzig, Germany; ^4^Department of Medicine, Health and Medical University Potsdam, Potsdam, Germany

**Keywords:** PLOD family, prognosis, tumor microenvironment, immune infiltration, immunotherapy, lower-grade glioma

## Abstract

Lower-grade glioma (LGG) is a group of tumors arising from the cells of the central nervous system. Although various therapy interventions are used, the prognosis remains different. Novel biomarkers are needed for the prognosis of disease and novel therapeutic strategies in LGG. The procollagen-lysine, 2-oxoglutarate 5-dioxygenase (PLOD) family contains three members and is related to multiple cancers, yet it was not investigated in LGG. Data from the Chinese Glioma Genome Atlas (CGGA) and The Cancer Genome Atlas (TCGA) cohorts were used to analyze the role of PLOD in LGG. As the PLOD family is involved in processes, such as tumor formation and cancer metastasis, we focused on its relationship to the tumor microenvironment (TME) in LGG. A high expression of the PLOD family relates to poor prognosis and high infiltration of immune cells within the TME. The expression level of the PLOD family might become a novel biomarker for prognosis and is a potential target for individual treatment decisions in LGG.

## Introduction

Lower-grade glioma (LGG) is a group of tumors arising from the cells of the central nervous system. In 2016, the World Health Organization (WHO) reclassified LGG using typical histopathological features by crucial markers, such as isocitrate dehydrogenase 1 (IDH1), which disclosed the fact that the biological markers were closely associated with the prognosis of LGG (Louis et al., 2016). The current LGG treatment involves different methods, including surgery and radiotherapy as well as chemotherapy (Van Den Bent, 2015; Hervey-Jumper and Berger, 2016; Jin et al., 2018; Tom et al., 2019; Wang and Mehta, 2019). While intensive therapeutic interventions are applied to LGG, the outcome remains different. The cellular alterations of the glioma and its surrounding tissue build the tumor microenvironment (TME) (Müller et al., 2017). Neoplastic and non-neoplastic cells, such as cancer-associated fibroblasts and immune cells, both of which are involved in tumor formation, progression, and especially the response to treatment, are important modulators of the TME (Tan et al., 2020). Most of the non-neoplastic cells are tumor-associated immune cells. However, the brain is not easily infiltrated by the immune system due to the blood-brain barrier (BBB). The adaptation of infiltrating immune cells, such as monocytes and T cells to brain tissue or neoplastic tissue, is essential to further understand tumor development and progression. The immunological response to growth factors and cytokines created by neoplastic cells might determine disease progression (Hambardzumyan et al., 2015). Resident immune cells and infiltrated immune cells might determine the disease-associated microenvironment in the brain. While focusing on the TME, rather than glioma cells themselves, recent literature identified the TME as a superior way to improve the prognosis of LGG (Müller et al., 2017; Jin et al., 2018).

One of the main components of the TME is the extracellular matrix (ECM). Especially, collagen plays a crucial role in the physiological tissue function and tumor formation (Qi and Xu, 2018). Genetic defects affect the biosynthesis, assembly, post-translational modification, and secretion of collagen, and can lead to collagen-related diseases or even cancer (Heikkinen et al., 1994; Vahidnezhad et al., 2019; Li S. S. et al., 2020). Procollagen-lysine and 2-oxoglutarate 5-dioxygenases (PLODs) catalyze lysyl hydroxylase (LH), participating in the process of covalent cross-links and collagen glycosylation. Deposition and cross-linking of collagen within the ECM offer a chemical and physical support for tumor formation and proliferation (Qi and Xu, 2018). Three members of the PLOD family (i.e., PLOD1, PLOD2, and PLOD3) leading to cancer progression and metastasis when dysregulated have already been identified (Jiang et al., 2020; Li S. S. et al., 2020). It has recently been recognized that PLOD1 is overexpressed in glioma (Tian et al., 2021). Our previous work demonstrated that PLOD3 is also highly expressed in LGG with a poor prognosis (Gong et al., 2021). Here, we focused on the regulation of the TME and prognostic functions of the PLOD family in LGG.

## Materials and Methods

### Datasets

The RNA-Seq data and clinical information of 431 patients with LGG in the discovery cohort were obtained from the Chinese Glioma Genome Atlas (CGGA^1^) database (Zhao et al., 2021). As an independent validation cohort, RNA-Seq data and clinical information of 510 patients with LGG were obtained from The Cancer Genome Atlas (TCGA) database^2^ (Supplementary Table 1). Samples that did not have complete survival information and paired RNA-Seq data were excluded from this study (174 of 605 samples from the CGGA dataset and 3 of 513 samples from the TCGA database were excluded). All data have been normalized and log_2_(*x* + 1) transformed.

### Gene Expression Profiling Interactive Analysis 2

Gene expression profiling interactive analysis 2 (GEPIA2)^3^ is a website tool that can be used for analyzing the gene expression distinctions between tumor and normal tissues based on TCGA and Genotype-Tissue Expression (GTEx) databases. In this study, the GEPIA2 tool was used to compare the expression levels of PLOD family members between 518 LGG tumor tissues and 207 normal brain tissues. In addition, the top 100 PLOD-related genes were also obtained in GEPIA2. GEPIA2 tool was also used to obtain the expression of PLOD family members in glioblastoma (GBM) compared with normal brain tissues. The survival analysis module of GEPIA2 was employed to acquire the data of overall survival (OS) and disease-free survival (DFS) in GBM.

### Human Protein Atlas Analysis

Human Protein Atlas (HPA^4^) is a project that focused on exploring the human protein in cells, tissues, and organs based on the combination of several omics technologies. In this study, the protein expression level of all the three PLOD family members and immunological cells (e.g., CD3 and CD68) was retrieved from the LGG tumor tissues and correlative normal tissues of the HPA dataset.

### cBioPortal Analysis

The cBioPortal^5^ is a tool for the analysis of genomic datasets. In this study, all the PLOD family members were typed into the cBioPortal website to obtain the genetic alternation data based on the TCGA dataset.

### Gene Set Enrichment Analysis

Gene set enrichment analysis (GSEA) software (version 4.1.0) was used to perform GSEA by using the HALLMARK gene set. The significance threshold was set to *p* < 0.05, and false discovery rate (FDR) was set to <0.25.

### Gene Enrichment Analysis

In this study, the Search Tool for the Retrieval of Interacting Genes/Proteins (STRING)^6^ database was used to obtain the top 50 PLOD-interacted proteins and construct the protein-protein interaction (PPI) network. Combining the top 50 PLOD-interacted genes and the top 100 PLOD-related genes, the “clusterProfiler” R package was used to perform the Kyoto Encyclopedia of Genes and Genomes (KEGG) pathway analysis and the Gene Ontology (GO) enrichment analysis.

### Immune Infiltration Analysis

The R package “GSVA” was used to perform a single-sample GSEA (ssGSEA) to quantify the relative abundance of 28 previously defined immune cells (Charoentong et al., 2017). The R package “ESTIMATE” was used to calculate three scores, namely, ImmuneScore (positively correlated with the level of immune cell infiltration in the tumor), StromalScore (positively correlated with the level of stroma cell in the tumor), and ESTIMATEScore (negatively correlated with tumor purity) (Yoshihara et al., 2013). The TIMER tool^7^ was used to obtain six immune cells infiltration in GBM.

### Patient-Derived Glioma Tissue

Patient-derived glioma tissue was obtained from the University Hospital Leipzig. Glioma tissue from nine different patients was stained with 3,3′-diaminobenzidine (DAB; Sigma Aldrich, St. Louis, MO, United States) tablets as described previously (Brückner et al., 2021). The following primary antibodies were used in this study: CD3 antibody (MCA1477, lot no. 149500B, 1:200 dilution) and CD68 antibody (M0876, lot no. 20043031, 1:500 dilution).

### Statistical Analysis

Based on multivariate analysis, the risk score model, namely, PLODscore was calculated. This type of model was already published in our previous work and in high-grade glioma research (De Tayrac et al., 2011; Wen et al., 2021; Wu et al., 2021). The Spearman’s correlation analysis was used to calculate the correlations between PLODscore and the level of 28 immune cells infiltration, ImmuneScore, StromalScore, and ESTIMATEScore. The Kaplan–Meier (KM) curves were used to estimate the difference in survival between two groups, and significance was calculated using a log-rank test. The receiver operating characteristic (ROC) curve and the Harrell’s concordance index were used to assess the predictive value of the risk model. Differences between the two groups were calculated using the unpaired Student’s *t*-test or Wilcoxon rank-sum test. Comparisons of more than two groups were calculated using the Kruskal-Wallis test. Multivariate Cox regression was performed to evaluate the independent risk factors on OS. All statistical calculations were performed using R software (version 4.0.3; R Foundation for Statistical Computing, Vienna, Austria), and *p* < 0.05 was considered statistically significant.

## Results

### The Alternation and Expression of Procollagen-Lysine, 2-Oxoglutarate 5-Dioxygenase Family Members at Transcription and Translation Level

**FIGURE 1:**
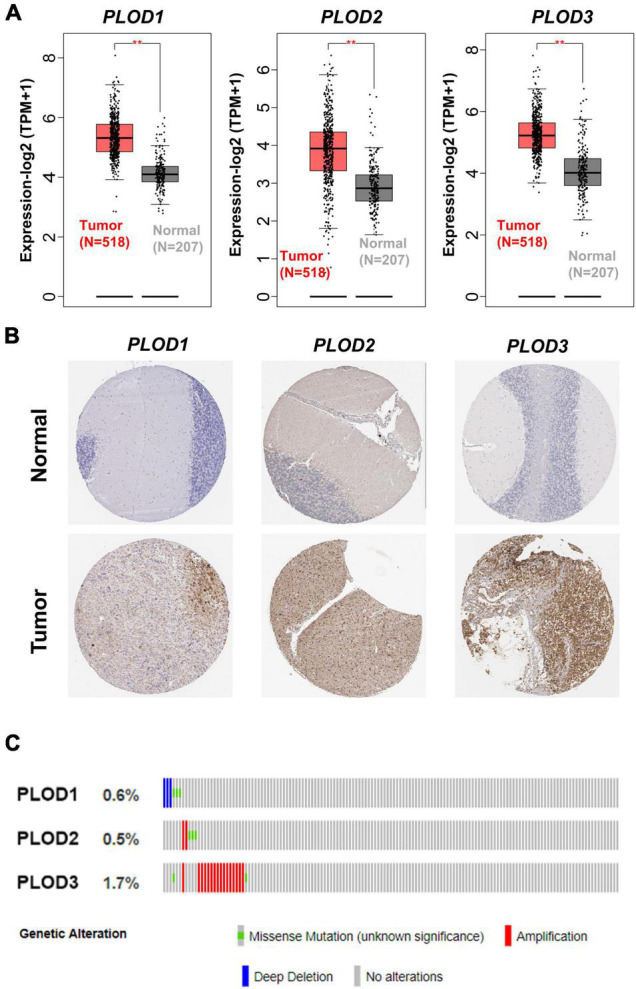
The alternation of procollagen-lysine, 2-oxoglutarate 5-dioxygenase (PLOD) family members and the expression of PLODs at transcription and translation level in low-grade glioma (LGG). **(A)** Gene expression profiling interactive analysis 2 was used to harvest the boxplot results of the expressions of PLODs family members between cancer tissues and correlative normal tissue at transcriptional level. **(B)** Based on the Human Protein Atlas database, the expression of PLODs was obtained at protein level according to the immunohistochemistry staining. **(C)** The genetic alternations of PLODs were achieved by cBioPortal. **p < 0.01.

In total, 518 LGG tumor tissues from the TCGA database and 207 correlative normal brain tissues from the GTEx database were included based on GEPIA2. PLOD1, PLOD2, and PLOD3 were highly expressed in LGG tumor tissues compared to normal tissues (Figure 1A, *p* < 0.01). The determination of the difference in protein levels was also achieved by using the HPA database. The expression of all PLOD family members was higher in LGG tumor tissues than that of the normal tissues by immunohistochemical staining (Figure 1B). The cBioPortal was used to obtain the alternations of PLOD1, PLOD2, and PLOD3, including the alternation types and the correlative rate. The alternation rate for PLOD1, PLOD2, and PLOD3 accounted for 0.6, 0.5, and 1.7%, respectively. The amplification occupied the most part of PLOD3 alternation (Figure 1C). In addition, the PLOD family members were also highly expressed in GBM (Supplementary Figure 1A).

### The Expression of Procollagen-Lysine, 2-Oxoglutarate 5-Dioxygenase Family Members in Correlation to Different Clinical Parameters

**FIGURE 2:**
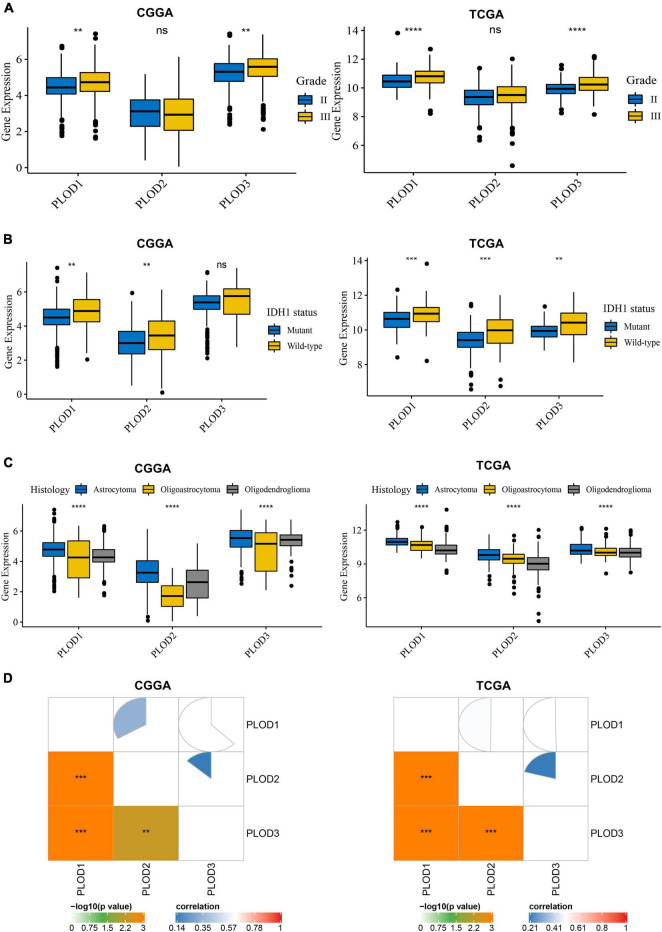
Procollagen-lysine, 2-oxoglutarate 5-dioxygenases (PLOD) expressions between diverse clinical stratifications and the relationship of expression levels among PLODs family members in lower-grade glioma (LGG) based on the datasets of Chinese Glioma Genome Atlas (CGGA) and The Cancer Genome Atlas (TCGA). **(A)** PLODs expression between different grades in LGG. **(B)** PLODs expression based on the status of is citrate dehydrogenase 1 (IDH1). **(C)** PLODs expression based on the histology of LGG. **(D)** The relationship of expression levels among the PLODs family members (e.g., PLOD1, PLOD2, and PLOD3) based on CGGA and TCGA databases. **p < 0.01, ***p < 0.001, ****p < 0.0001. ns, not significant.

Data from CGGA and TCGA datasets were analyzed. Diverse clinical parameters were considered in this analysis, including LGG grade, IDH1 status, histology of LGG, age, and gender. Figure 2A shows that the expression level of PLOD1 and PLOD3 is higher in grade III than grade II samples but does not alter PLOD2 levels. In addition, the expression level of PLOD1 and PLOD2 was higher in IDH1 wild-type samples than IDH1 mutant samples in both datasets, while PLOD3 was only significantly enhanced in the IDH1 wild-type status group of the TCGA dataset (Figure 2B). According to the classification of TCGA and CGGA databases, three main pathological subtypes were included in this study. Among the different types of histology, the expression of all PLOD family members was higher in astrocytoma than in oligoastrocytoma and oligodendroglioma of CGGA and TCGA datasets (Figure 2C). Supplementary Figure 2 indicates that there are no significant differences in PLOD1-3 expressions in different age and sex groups. Furthermore, the expression between the PLOD family was positively correlated with LGG (Figure 2D).

### Kyoto Encyclopedia of Genes and Genomes Pathway and Gene Ontology Enrichment Analyses for Procollagen-Lysine, 2-Oxoglutarate 5-Dioxygenase Family Members

**FIGURE 3:**
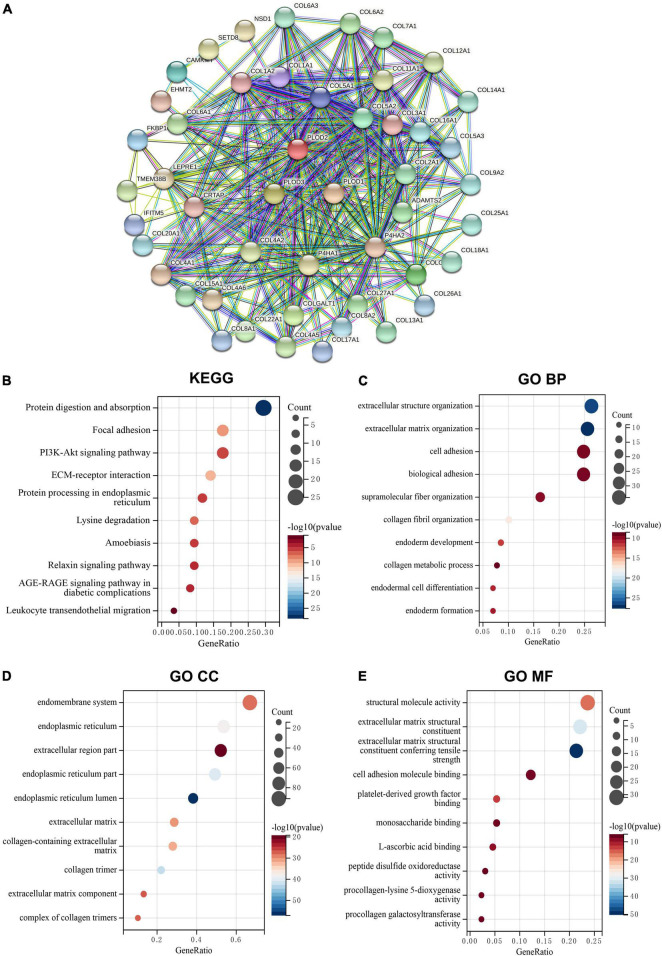
The protein-protein interaction (PPI) networks, Kyoto Encyclopedia of Genes and Genomes (KEGG) pathway, and Gene Ontology (GO) enrichment analysis for PLODs family members in lower-grade glioma (LGG). **(A)** Search Tool for the Retrieval of Interacting Genes/Proteins tool was used to obtain the PPI networks for PLODs. **(B)** KEGG pathway of PLODs. **(C)** GO enrichment of biological process for PLODs. **(D)** GO enrichment of cellular component for PLODs. **(E)** GO enrichment of molecular function for PLODs.

The top 50 PLOD-interacting genes were identified, and a PPI network was constructed based on behalf of the STRING tool, as shown in Figure 3A. In Supplementary Table 2, the top 100 PLOD-related genes are displayed. By combining PLOD-interacting genes with PLOD-related genes, the KEGG pathway and GO enrichment analyses were performed. According to the KEGG pathway analysis, these genes are involved not only in carcinogenesis-related pathways, such as focal adhesion and ECM-receptor interaction, but also in immune-related pathways, such as leukocyte migration (Figure 3B). The GO enrichment analysis indicates that PLOD-interaction and related genes are associated with ECM organization, cell adhesion, biological adhesion, and endodermal cell differentiation in the biological process (BP) section (Figure 3C). Further analysis revealed a significant relationship of the PLOD family with the endomembrane system and the ECM components of the cellular component (CC) section. Analysis of the molecular function (MF) section revealed the components of the ECM to be of relevance, as shown in Figures 3D,E.

### Overall Survival Analysis Based on the Expression of Procollagen-Lysine, 2-Oxoglutarate 5-Dioxygenase Family Members

**FIGURE 4:**
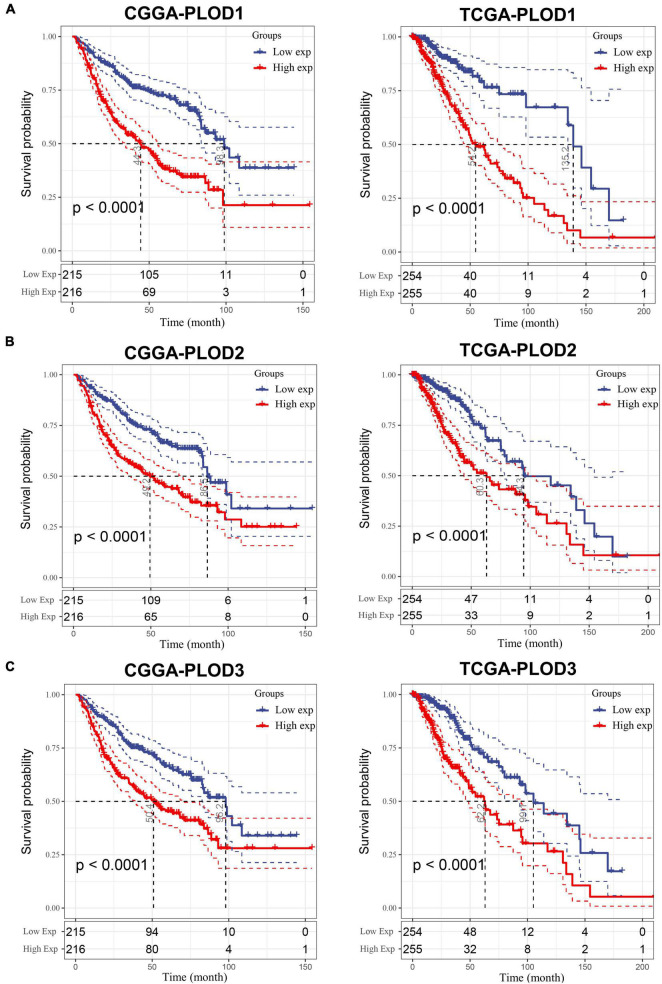
The survival analysis for the expression of procollagen-lysine, 2-oxoglutarate 5-dioxygenases (PLOD) members in lower-grade glioma (LGG). A cutoff value of 50–50% was applied and Kaplan–Meier (KM) curve was used to analyze and visualize the survival analysis based on Chinese Glioma Genome Atlas (CGGA) and The Cancer Genome Atlas (TCGA) datasets. **(A)** Correlation between survival period and PLOD1 expression level in LGG. **(B)** Correlation between survival period and PLOD2 expression level in LGG. **(C)** Correlation between survival period and PLOD3 expression level in LGG.

The data from CGGA and TCGA datasets were analyzed to explore the correlation of the expression of PLOD family members with the OS in LGG. The expression median separates the high and low expression of the PLOD family members, and the KM curves visualize the survival data, as shown in Figure 4. A high expression of all PLOD family members was correlated with worse OS in both datasets compared to a low expression. In addition, the overexpression of PLOD1 showed a correlation to poor prognosis in GBM, while the other PLOD family members were not linked to prognosis in GBM (Supplementary Figure 1).

### Establishment of PLODscore for Lower-Grade Glioma Prognostic Prediction

The gathered results suggest that the investigated PLOD members are related to tumorigenesis and development of LGG. To further quantify the expression pattern of PLOD family members in individual patients and to construct a PLOD-related gene signature, the multivariate Cox regression model was used to establish a scoring system based on the CGGA dataset, termed PLODscore. Specifically, the PLODscore was used to calculate the following formula:


$${\text{PLOD}}_{\text{score}}=\mathrm{PLOD1}.\text{exp}\times 0.31628+\mathrm{PLOD2}.\text{exp}$$

×0.30841+PLOD3.exp×0.11836.

**FIGURE 5:**
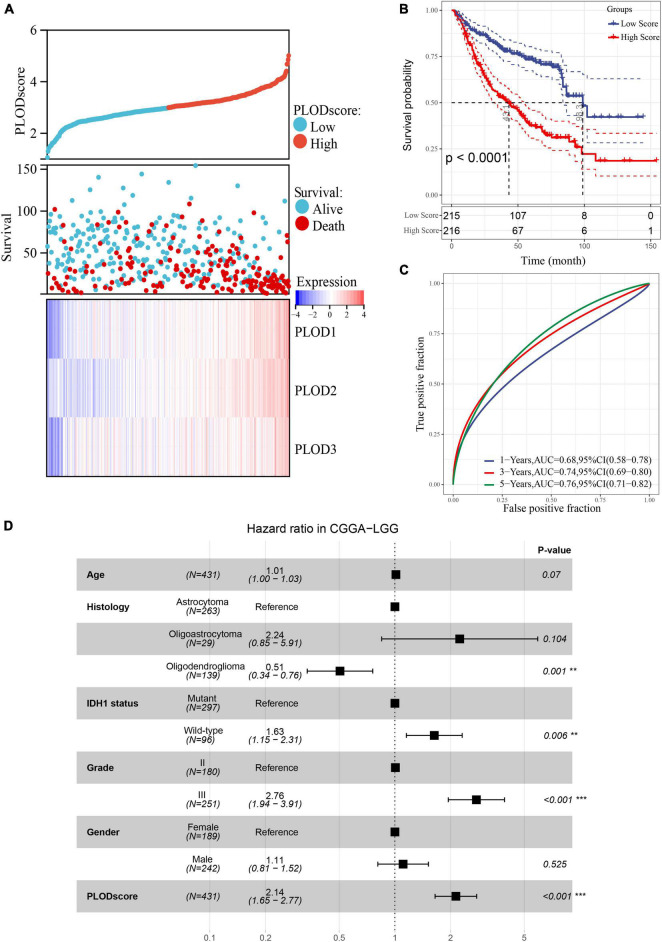
The association of PLODscore with the prognosis in lower-grade glioma (LGG) based on Chinese Glioma Genome Atlas (CGGA) dataset. **(A)** PLODscore, survival status, and heatmap of mRNA expression of the PLODs members. **(B)** Kaplan–Meier (KM) curve. **(C)** Time-dependent survival receiver operating characteristic analysis. **(D)** Multivariate statistics for procollagen-lysine, 2-oxoglutarate 5-dioxygenase (PLOD) score in LGG. **p < 0.01, ***p < 0.001.

After calculating the PLODscore for each sample, all samples in the CGGA cohort were divided into high and low PLODscore groups using the median as the threshold. As shown in Figure 5A, the high PLODscore group had a lower survival rate and higher PLOD expressions. The KM curve indicated that the OS of patients with a high PLODscore group was significantly reduced compared to the low PLODscore group (Figure 5B). Similar outcome could also be obtained if the PLODscore was quartered (Supplementary Figure 4). The area under the ROC curve (AUC) for 1, 3, and 5 years was 0.68, 0.74, and 0.76, respectively (Figure 5C). In addition, multivariate Cox regression analysis suggested that the PLODscore was an independent risk factor in LGG (Figure 5D). All the above results were independently validated in the TCGA cohort (Supplementary Figures 3, 4). Nomogram was established based on the independent risk factors in the CGGA cohort, and the decision curve analysis (DCA) indicates that the nomogram has a good predictive performance (Supplementary Figure 5) and the Harrell’s concordance index (Supplementary Table 3).

### Cellular and Molecular Characteristics of PLODscore

**FIGURE 6:**
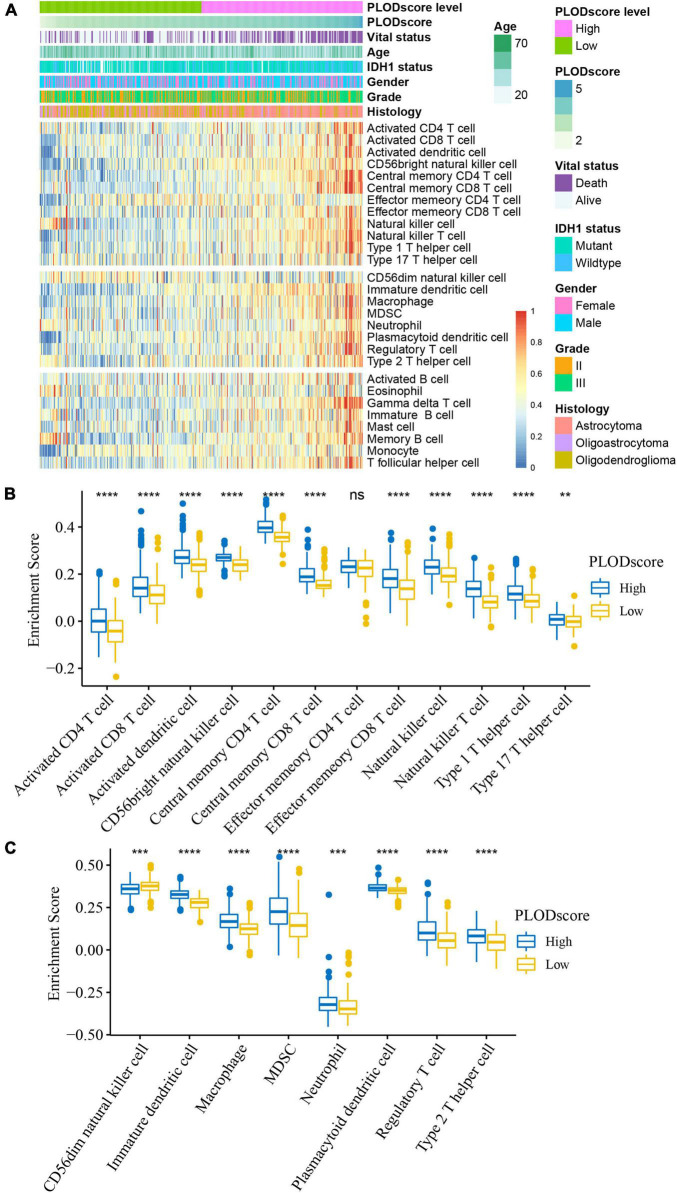
Cellular characteristics of PLODscore based on Chinese Glioma Genome Atlas (CGGA) cohort. **(A)** Multivariate statistics for PLODscore, including vital status, IDH1 status, gender, grade of lower-grade glioma (LGG), histology of LGG, and the heatmap of 28 previously reported immune cell signature scores. **(B)** The relationship between PLODscore and enrichment score of immunostimulatory cells. **(C)** The relationship between PLODscore and enrichment score of immunosuppressive cells. **p < 0.01, ***p < 0.001, ****p < 0.0001. ns, not significant.

Tumor response to immunotherapy is largely dependent on the TME (Quail and Joyce, 2017). Therefore, we investigated the potential role of PLOD family members within the TME of LGG. The staining from the HPA dataset and the patient-derived glioma tissues showed that there is a significant number of CD3 positive and CD68 positive cells in all stages of glioma tissue (Supplementary Figure 6 and Supplementary Table 4). However, the expression varies greatly within the patient samples. Using ssGSEA, we inferred the enrichment score of 28 previously reported immune cells of the CGGA cohort. The immune cell components between the high and low PLODscore groups were significantly different. Except for CD4 memory effector cells and CD56 natural killer cells, patients with a low PLODscore showed lower infiltration levels of most immune cells (Figures 6A–C). Similar results were found in the TCGA dataset (Supplementary Figure 7).

**FIGURE 7:**
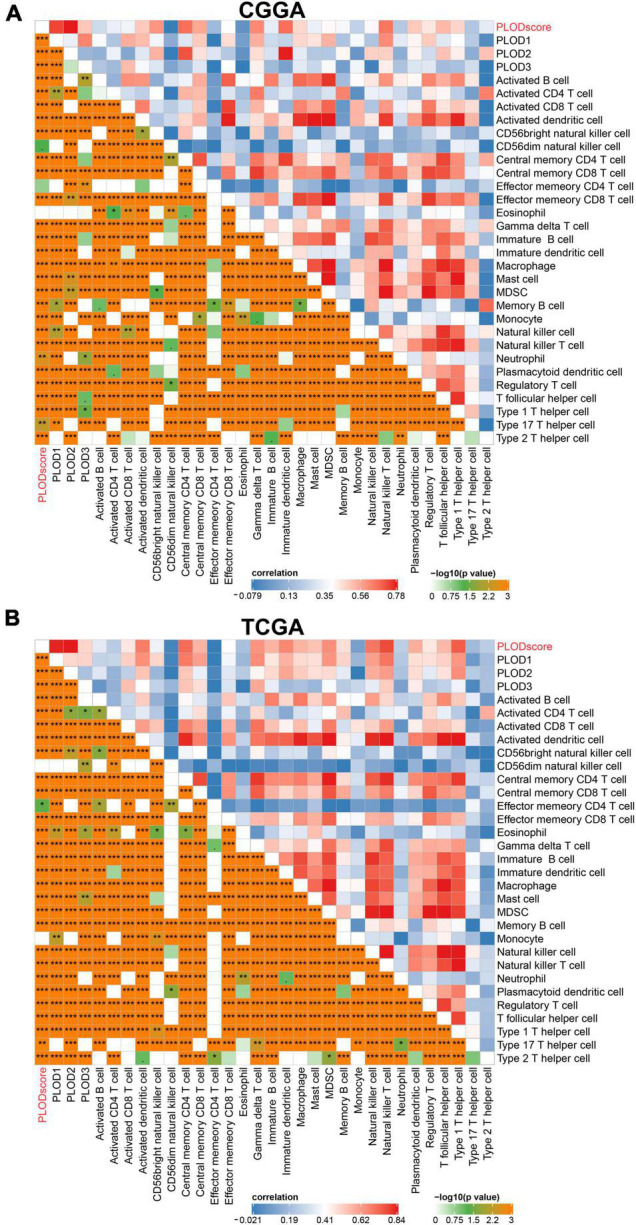
The immune landscape of procollagen-lysine, 2-oxoglutarate 5-dioxygenases (PLOD) in lower-grade glioma (LGG). The immune landscape of correlation between PLODscore, PLODs members, and immune cells according to the **(A)** Chinese Glioma Genome Atlas (CGGA) cohort and **(B)** The Cancer Genome Atlas (TCGA) dataset. *p < 0.05, **p < 0.01, and ***p < 0.001.

**FIGURE 8:**
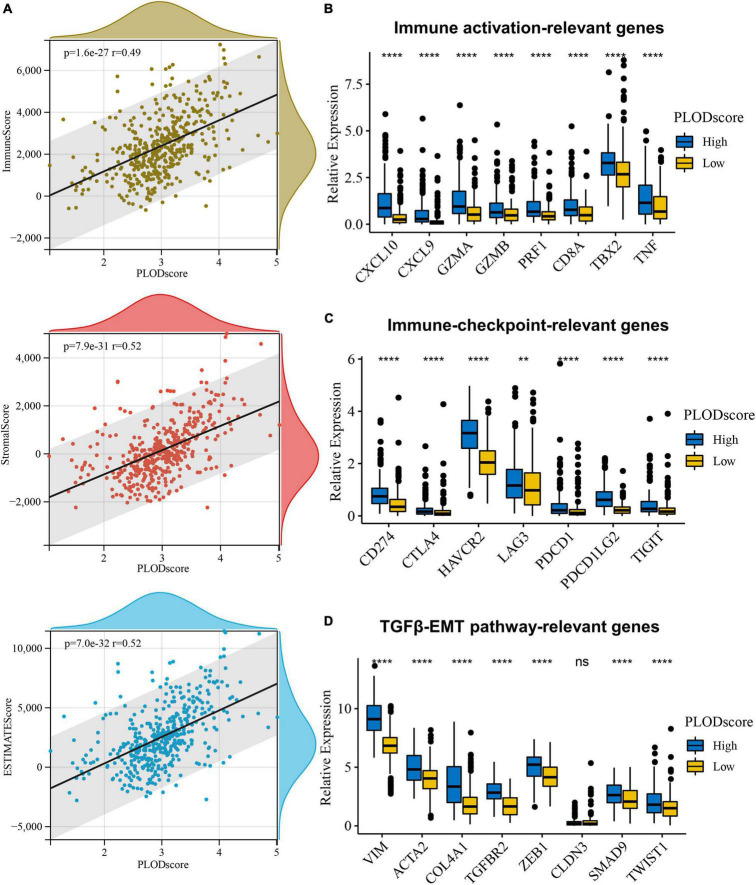
The overview of PLODscore with immune infiltration according to Chinese Glioma Genome Atlas (CGGA) database. **(A)** The association of PLODscore with ImmuneScore, StromalScore, and ESTIMATEScore. **(B)** The relationship of PLODscore to immune activation-relevant genes, **(C)** immune-checkpoint-relevant genes, and **(D)** transforming growth factor-β/epithelial-mesenchymal transition pathway-relevant genes. **p < 0.01, ****p < 0.0001. ns, not significant.

Furthermore, the PLODscore was positively correlated with the infiltration levels of multiple immunostimulatory cells (e.g., natural killer T cells) and immunosuppressive cells (e.g., regulatory T cells) by correlation analysis (Figures 7A,B). Patients with a high PLODscore have a “hotter” but more immunosuppressed TME, which implies that the PLODscore could quantify the TME pattern of individual patients. In addition, the expression of the PLOD family members could be related to infiltrating immune cells, such as B cells, T cells, and macrophages (Supplementary Figure 8). To further test this inference, we calculated the estimated scores for patients with LGG based on the study by Yoshihara et al. (2013). It was found that the PLODscore was positively related to the ImmuneScore, StromalScore, and ESTIMATEScore (Figure 8A), which suggests that the high PLODscore group has higher immune and stromal cell infiltration. In addition, we analyzed the expression of selected cytokine and chemokine mRNAs. We considered CXCL10, CXCL9, GZMA, GZMB, PRF1, CD8A, TBX2, and TNF as immune-activating transcripts; IDO1, CD274, HAVCR2, PDCD1, CTLA4, LAG3, and PDCD1LG2 as immune checkpoint transcripts; and VIM, ACTA2, COL4A1, TGFBR2, ZEB1, CLDN3, SMAD9, TWIST1, and TGRB1 as transforming growth factor (TGF)β/epithelial-mesenchymal transition (EMT) pathway transcripts (Mariathasan et al., 2018; Zeng et al., 2019). The high PLODscore group had enhanced expression levels of immune activation-relevant genes, immune-checkpoint-relevant genes, and TGFβ/EMT pathway-relevant genes (Figures 8B–D). These results demonstrated that patients with high PLODscore are characterized by immune activation and stromal activation with significant immunosuppression. Similar results were also obtained in the TCGA dataset (Supplementary Figure 9).

### Gene Set Enrichment Analysis of PLODscore

**FIGURE 9:**
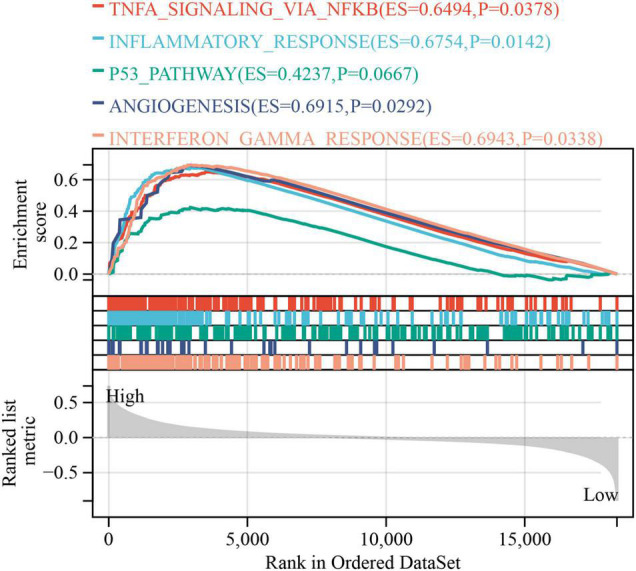
Gene set enrichment analysis. The relationship of PLODscore with biological processes and signal transduction pathways using the HALLMARK gene set.

The GSEA was used to explore the potential BPs and signal transduction pathways related to PLODscore. A high PLODscore is related to carcinogenesis-related pathways, including angiogenesis and P53 pathway, as well as immune activation-related pathways, including inflammatory response and interferon-gamma (IFNγ) response (Figure 9). This is consistent with our previous results that patients with high PLODscore have a poor prognosis together with a “hot” but immunosuppressed TME.

## Discussion

Our previous work indicated that PLOD3 overexpression is linked to poor prognosis in LGG and is associated with immune cell infiltration of the TME (Gong et al., 2021). In this study, a comprehensive analysis of the correlation between specific PLOD family members (e.g., PLOD1, PLOD2, and PLOD3) and LGG has been performed using the CGGA and TCGA datasets. These PLOD family members are suggested to be potential biomarkers for gastric cancer and hepatocellular carcinoma (Li S. S. et al., 2020; Yang et al., 2020). A further study has shown that PLOD1 is a predictive biomarker for LGG (Tian et al., 2021). Hence, we found that not only PLOD1 but also PLOD2 and PLOD3 were abundantly expressed in tumor tissues.

The PLOD catalyzes the LH, which is involved in the biosynthesis of collagen (Qi and Xu, 2018). Consequently, mutations of the PLOD family lead to connective tissue-related diseases, such as the Ehlers-Danlos and Bruck syndromes (Hjalt et al., 2001). During the period of connective tissue repair, the overexpression of the PLOD family might induce EMT and, consequently, contribute to tumor promotion. In EMT, the epithelial cells become unstable by losing their polarity and their adherence ability, and enhancing cellular migration and cellular invasion (Stone et al., 2016; Lee, 2019). This process takes place in important processes, such as wound healing, and can also lead to tumor formation (Suarez-Carmona et al., 2017). Supporting this hypothesis, the KEGG pathway and GO enrichment analysis correlate all three PLOD genes to alterations in focal and cellular adhesion and ECM-receptor interaction. The KEGG pathway analysis additionally showed that all PLOD genes are associated with leukocyte transendothelial migration, which might indicate the direct effect of PLOD in the TME. The IDH1 is a known biomarker for patients with glioma. Its mutation is regarded as beneficial, as epidermal growth factor receptor (EGFR) and loss of chromosome 10 are hardly found (Khan et al., 2017). Therefore, we tested the dependency of PLOD expression and the mutation status of IDH1. Enhanced PLOD1 and PLOD2 expression was found in the group, displaying wild-type IDH1. Although no significance was reached when compared to age and gender, the results fortified the suggestion that high PLOD expression is negatively linked to patient survival, as the expression of PLOD1 and PLOD3 is higher in grade III than grade II LGG (Miller et al., 2017). Still, PLOD3 could not be directly related to the IDH1 mutational status.

In this study, we created a PLODscore that was dependent on the expression of all three PLOD family members based on multivariate analysis. The defined PLODscore is negatively associated with the patient OS and serves as an independent prognostic factor for OS as well as tumor grade in LGG. Yet, it has to be shown whether the PLODscore has a prognostic significance for treatment response in all different glioma grades. In GBM, the PLODscore was, however, not applicable. Nevertheless, we could stain peripheral immune cells in GBM and LGG tissue, demonstrating relevant infiltration in individual tissues. Investigating the TME, a positive correlation with the PLODscore was observed, indicating a high potential relevance of the PLODscore in immunomodulatory therapies of LGG.

The TME consists of endothelial cells, fibroblasts, immune cells, and tumor cells (Quail and Joyce, 2013). However, as a tumor of the central nervous system, the TME of LGG has various distinguished features compared to peripheral tumor entities. Thus, almost all mentioned cells are found in glioma, but further unique CCs, such as microglia, astrocytes, and neurons, are also present (Wu et al., 2021). Fibroblasts are not present in the central nervous system; however, recent studies suggest that specific pericytes of the brain act as cancer-associated fibrocyte-like stromal cell population (Li M. et al., 2020). Here, we used patient-derived LGG and GBM samples to determine the amount of immune cell infiltration to affirm clinical relevance. Interestingly, CD3 and CD68 positive cells are observed to a higher extent only in patients with GBM and LGG, supporting the relevance of the TME together with personalized approaches in glioma (Supplementary Figure 6). Brain macrophages are the most abundant immune cell population in the TME of brain tumors. Even though its ontogenesis reveals a specialized, long-lasting macrophage population, peripheral macrophage invasion is likely to happen (Graeber et al., 2002; Shi and Pamer, 2011; Goldmann et al., 2016). The BBB is a cellular fence of endothelial cells and pericytes that needs to be conquered by immune cells or molecules to invade the brain parenchyma (Dyrna et al., 2013). The functional unit of the BBB is the so-called neurovascular unit (NVU) and protects the brain from immune invasion (Liu et al., 2018). However, the NVU loses its function in some diseases, such as Alzheimer’s disease (Lorger et al., 2019). Mice studies demonstrated the augmented effects of immune checkpoint inhibition in intracranial tumors when extracranial tumors were present (Volovitz et al., 2011; Taggart et al., 2018). Further studies based on animal models investigating multiple sclerosis demonstrated that immune cells, such as B cells, might activate CNS inflammation (Schnell, 1999; Magliozzi et al., 2007; Correale et al., 2017). However, it was shown that monocytes influence B cells to suppress CD8^+^ T cell activation and acquisition of an effector phenotype in GBM studies (Lee-Chang et al., 2019). Currently, antibodies against the intercellular adhesion molecule-1 were shown to reduce B cell invasion, demonstrating that B cells have the potential to be addressed in tumor progression (Jelcic et al., 2018; Comi et al., 2021). Hence, the integrity of the BBB is compromised by tumor formation or the progression of the parenchymal function needs to be preserved for the sake of neuronal survival (Weiss et al., 2009). Consequently, the TME of brain tumors is regarded to be immune-specialized.

Here, we used ssGSEA to investigate the PLODscore with immune cells of the TME in LGG. A positive correlation of the PLODscore with cells of the monocyte lineage and other cells determined as immunosuppressive was observed. Nevertheless, lymphoid cells, including CD4 positive and CD8 positive T cells, B cells, and NK cells, are also related to the PLODscore. A murine glioma study indicates that CD8 T cells alter depending on the microenvironment and might be differentially regulated in brain tissue (Masson et al., 2007). Our study demonstrated that the high PLODscore can be related to both an elevated T cell population and a high number of immunosuppressive cells, linking a high PLODscore to a hot but suppressive immune environment.

To further investigate this correlation, we used the method ESTIMATE, allowing for the prediction of stromal and immune fractions in tumor tissues (Yoshihara et al., 2013; Wen et al., 2021). The high PLODscore could be related to immune-activating genes (e.g., *CXCL10*, *CXCL9*, *TBX2*, and *TNF*) that are previously mentioned (Jelcic et al., 2018; Comi et al., 2021).

The correlation of the high PLODscore to immune checkpoint genes demonstrates the potential of immune checkpoint inhibition therapy in LGG. However, mouse models and clinical data demonstrate controversial results that might be resolved with adequate delivery systems (Galstyan et al., 2020; Khasraw et al., 2020). Further targetable genes of the TGFβ/EMT pathway are associated with the high PLODscore (Mikheev et al., 2018; Wang et al., 2018; Chandra et al., 2020; Suo et al., 2020; Han et al., 2021).

Although the mechanism of immune cell invasion is not yet understood in detail, evidence of patient-derived tissue samples provides important insight into the clinical relevance of immune cells spread throughout tumor tissues (Darmanis et al., 2017; Engelhardt et al., 2017; Gosselin et al., 2017; Venteicher et al., 2017; Wang et al., 2017). This analysis was based on two independent cohorts due to database limitations. Thus, the influence of brain-specific cells and the peripheral immune system to brain tumor formation and progression urges for further research (Galea et al., 2007; Engelhardt et al., 2017).

Taken together, this study shows the potential role of PLOD genes in LGG prognosis and its involvement in the TME of LGG. It is suggested that the regulation of PLOD genes and its products could be potential new targets for therapeutic interventions as well as for individual treatment decision in LGG.

## Data Availability Statement

The original contributions presented in the study are included in the article/Supplementary Material, further inquiries can be directed to the corresponding author.

## Ethics Statement

The studies involving human participants were reviewed and approved by the Ethics Committee of the Medical Faculty, University of Leipzig (#144/08-ek; 2019-07-04). The patients/participants provided their written informed consent to participate in this study.

## Author Contributions

SG, CW, NS, and SK: conception and design, data analysis and interpretation, and manuscript writing and revisions. SG, FK, JM, and CW: collection and assembly of data. All authors approved the final manuscript and accounted for all aspects of work, read, and agreed to the published version of the manuscript.

## Conflict of Interest

The authors declare that the research was conducted in the absence of any commercial or financial relationships that could be construed as a potential conflict of interest.

## Publisher’s Note

All claims expressed in this article are solely those of the authors and do not necessarily represent those of their affiliated organizations, or those of the publisher, the editors and the reviewers. Any product that may be evaluated in this article, or claim that may be made by its manufacturer, is not guaranteed or endorsed by the publisher.

## Footnotes

1
http://www.cgga.org.cn/

2
https://portal.gdc.cancer.gov/exploration

3
http://gepia2.cancer-pku.cn/

4
https://www.proteinatlas.org/

5
http://cbioportal.org

6
https://string-db.org

7
https://cistrome.shinyapps.io/timer
