# Supplementary material for: Procollagen-Lysine, 2-Oxoglutarate 5-Dioxygenase Family: Novel Prognostic Biomarkers and Tumor Microenvironment Regulators for Lower-Grade Glioma
Source: Front Cell Neurosci. 2022 Feb 18;16:838548. doi: 10.3389/fncel.2022.838548 (PMC8894330; doi:10.3389/fncel.2022.838548)
Supplement: Supplementary file 2 [file Data_Sheet_1.docx]

Supplementary Material

# Supplementary Figures and Tables

## Supplementary Figures


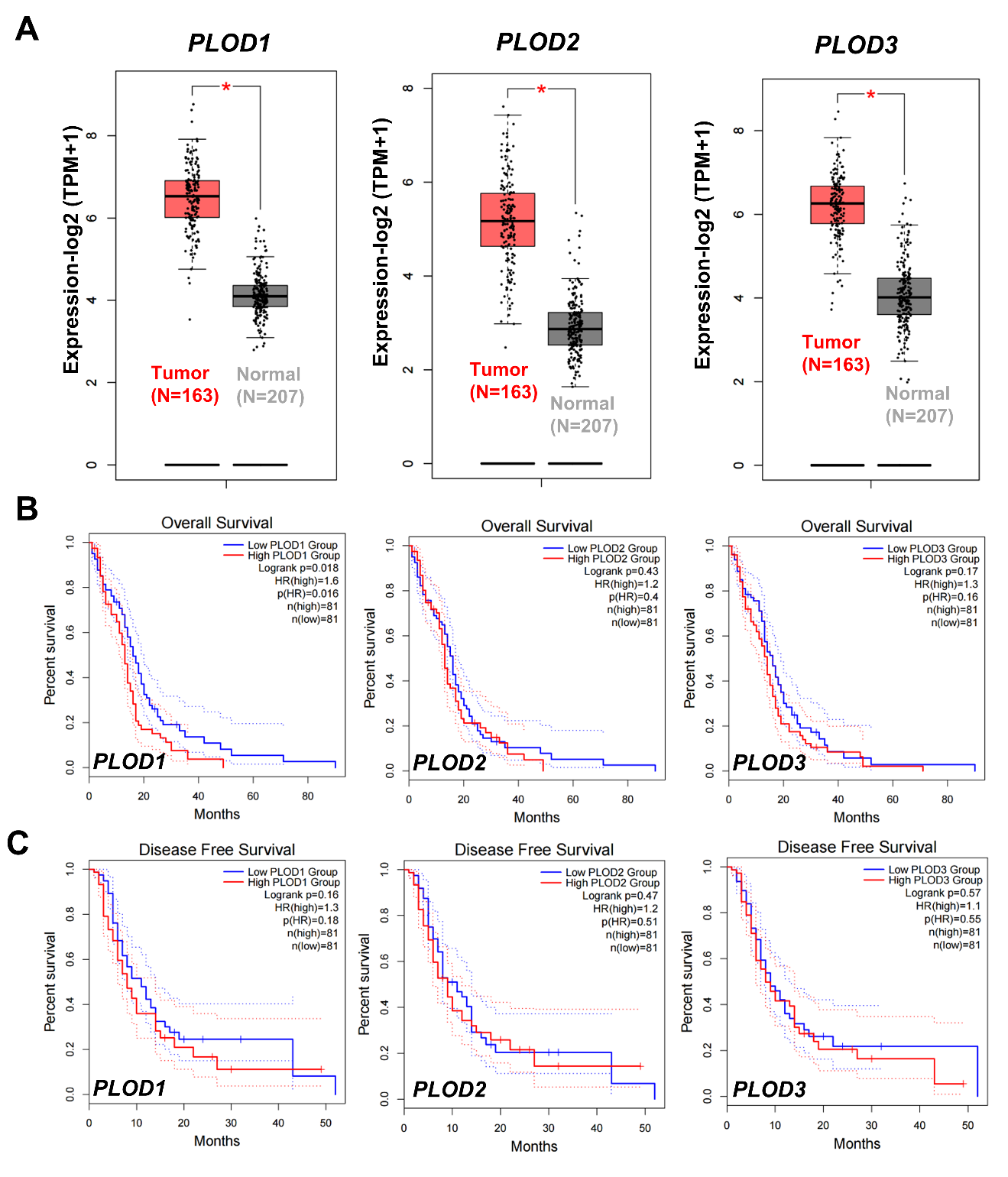


**Supplementary Figure S1. The expression of PLODs at Transcription level and the correlation between prognosis and expression of PLODs in GBM.** (A) GEPIA2 was used to harvest the Boxplot results of PLODs family members expressions between Cancer tissues and corelative Normal tissue at transcriptional level. (B) The correlation between Overall Survival and expression of PLODs. (C) The correlation between Disease Free Survival and expression of PLODs. *: p<0.05


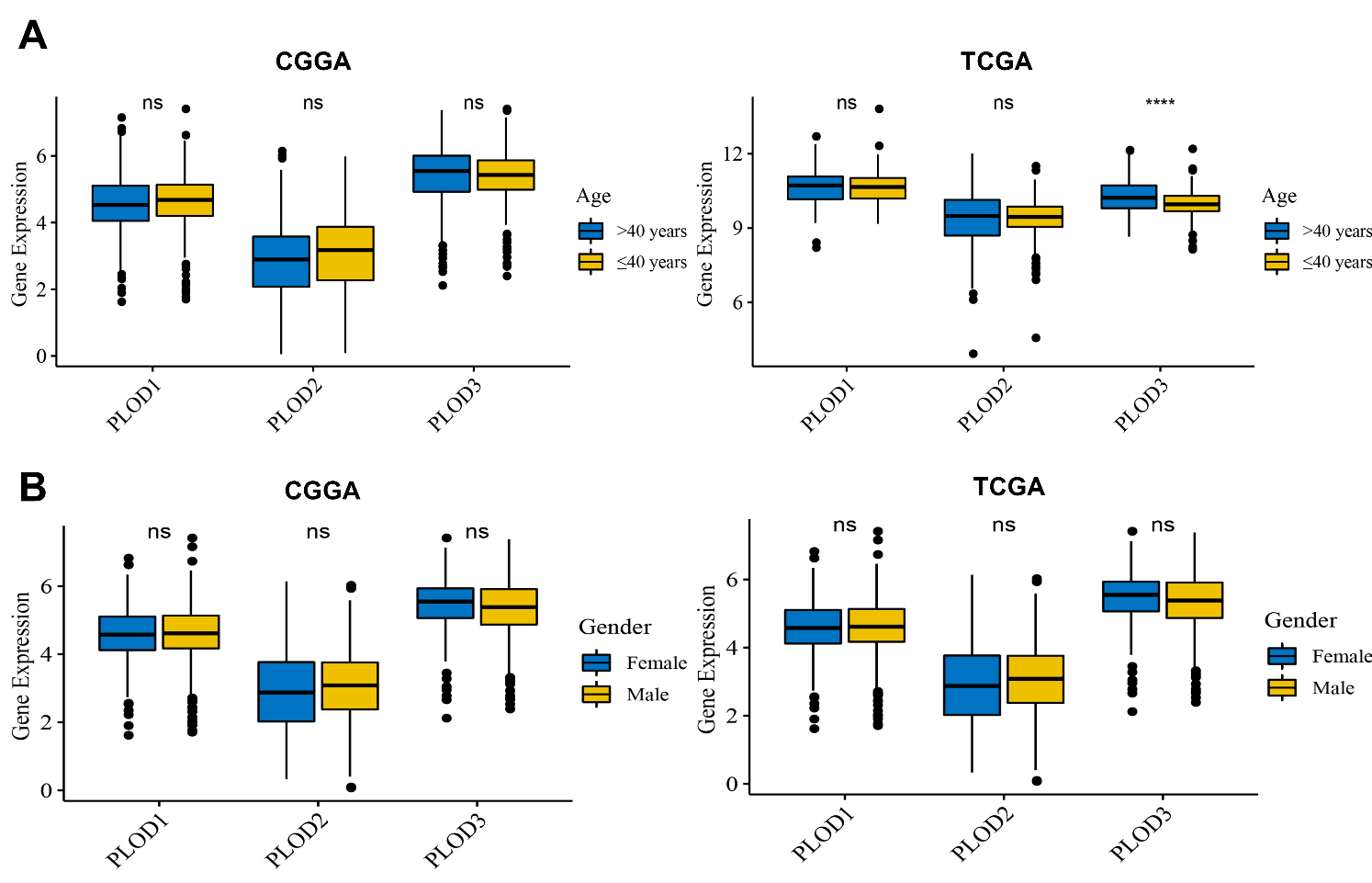


**Supplementary Figure S2.** PLODs expressions between different clinical stratifications and the relation of expression levels among PLODs family members in LGG based on the datasets of CGGA and TCGA. **(A)** PLODs Expression between different age in LGG. (B) PLODs Expression based on the statue of patients’ gender. ****: *p*＜0.0001, ns: not significant


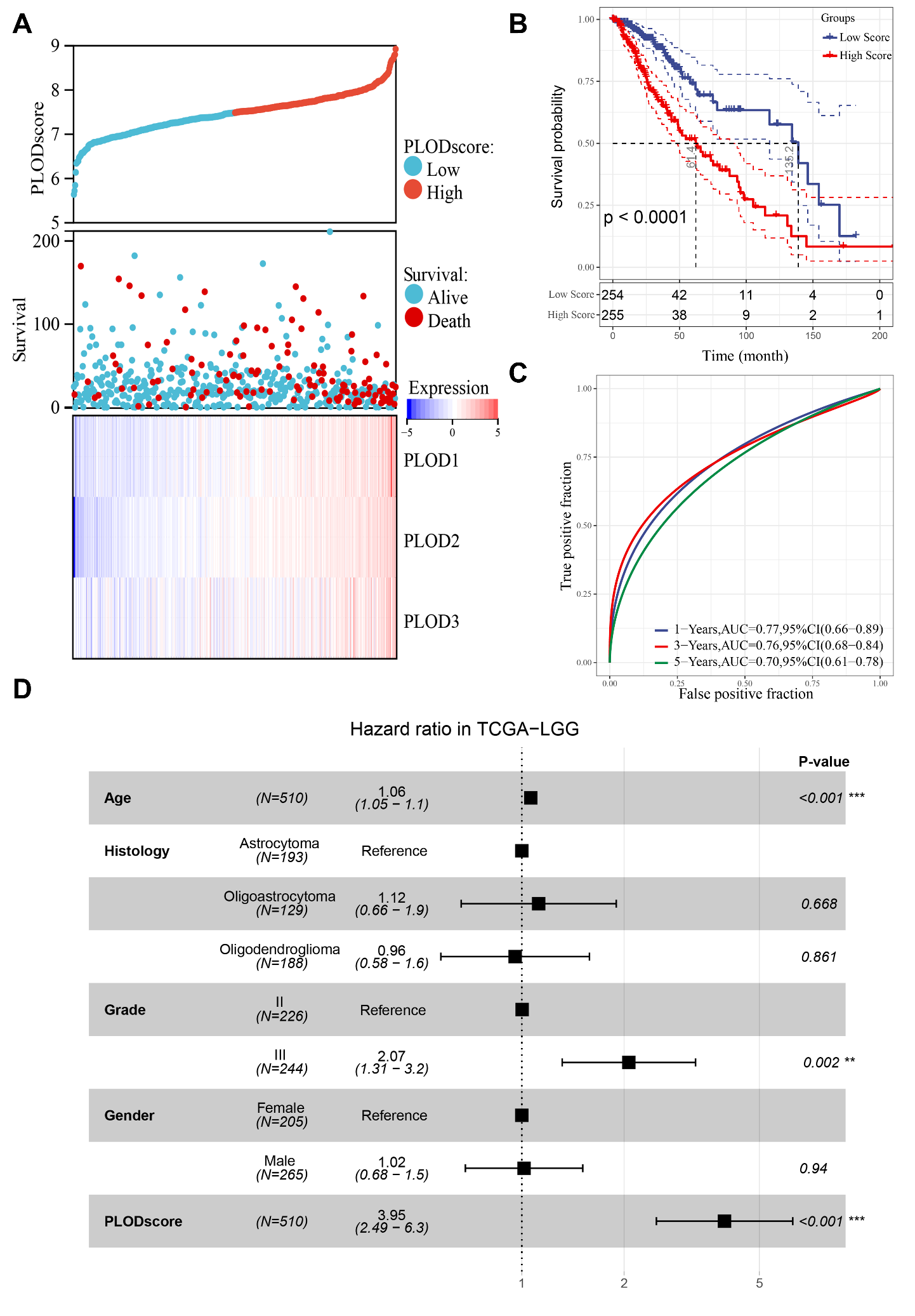


**Supplementary Figure S3.** The association of PLODscore with the prognosis in LGG based on TCGA dataset. **(A)** PLODscore, survival status and heatmap of mRNA expression of the PLODs members. **(B)** Kaplan–Meier curve. **(C)** Time-dependent survival ROC analysis. (**D**) Multivariate statistics for PLODscore in LGG. **: *p*<0.01, ***: *p*<0.001


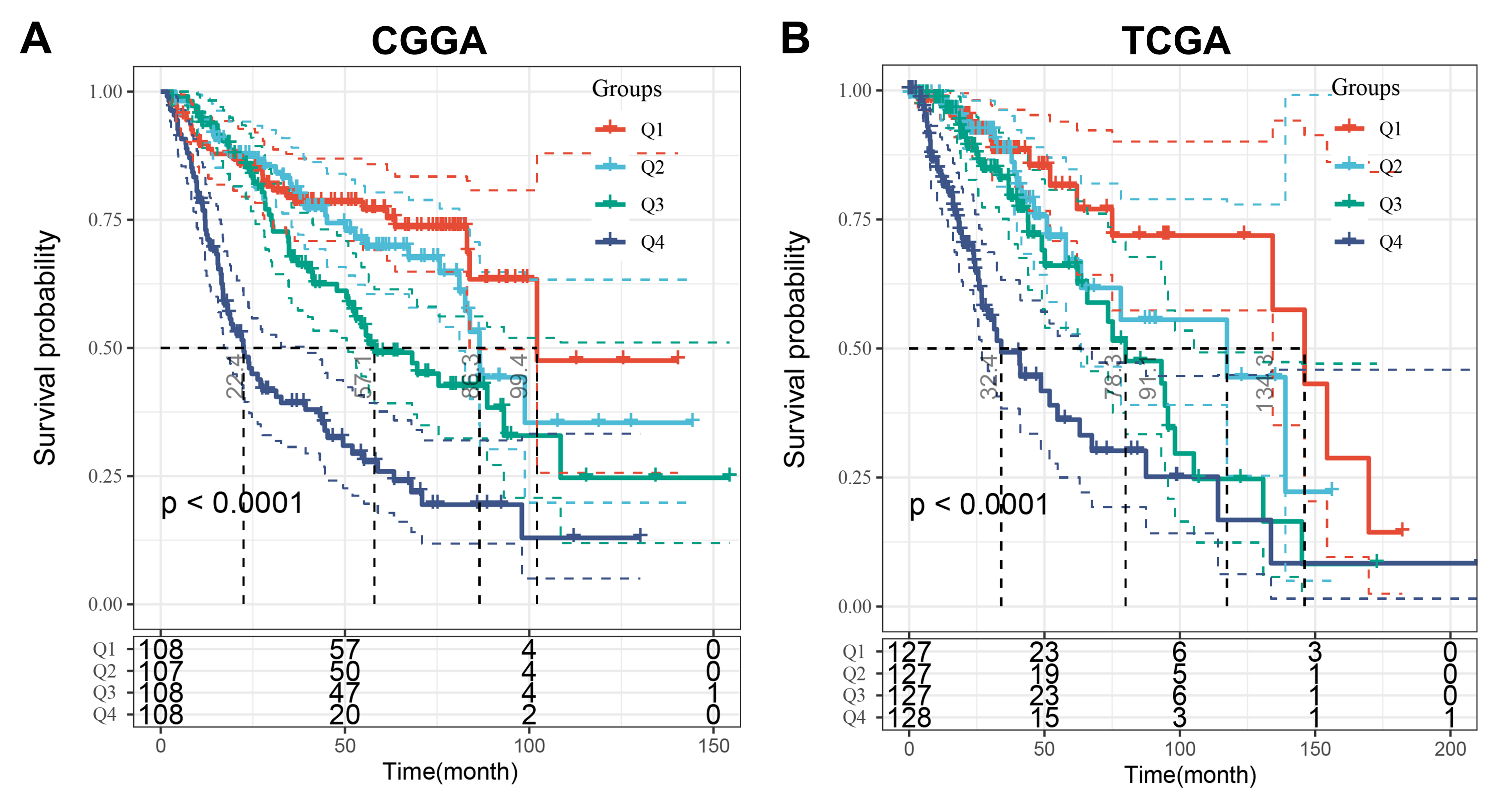


**Supplementary Figure S4.** The association of PLODscore with the overall survival in LGG **(A)** based on CGGA database and **(B)** TCGA database.


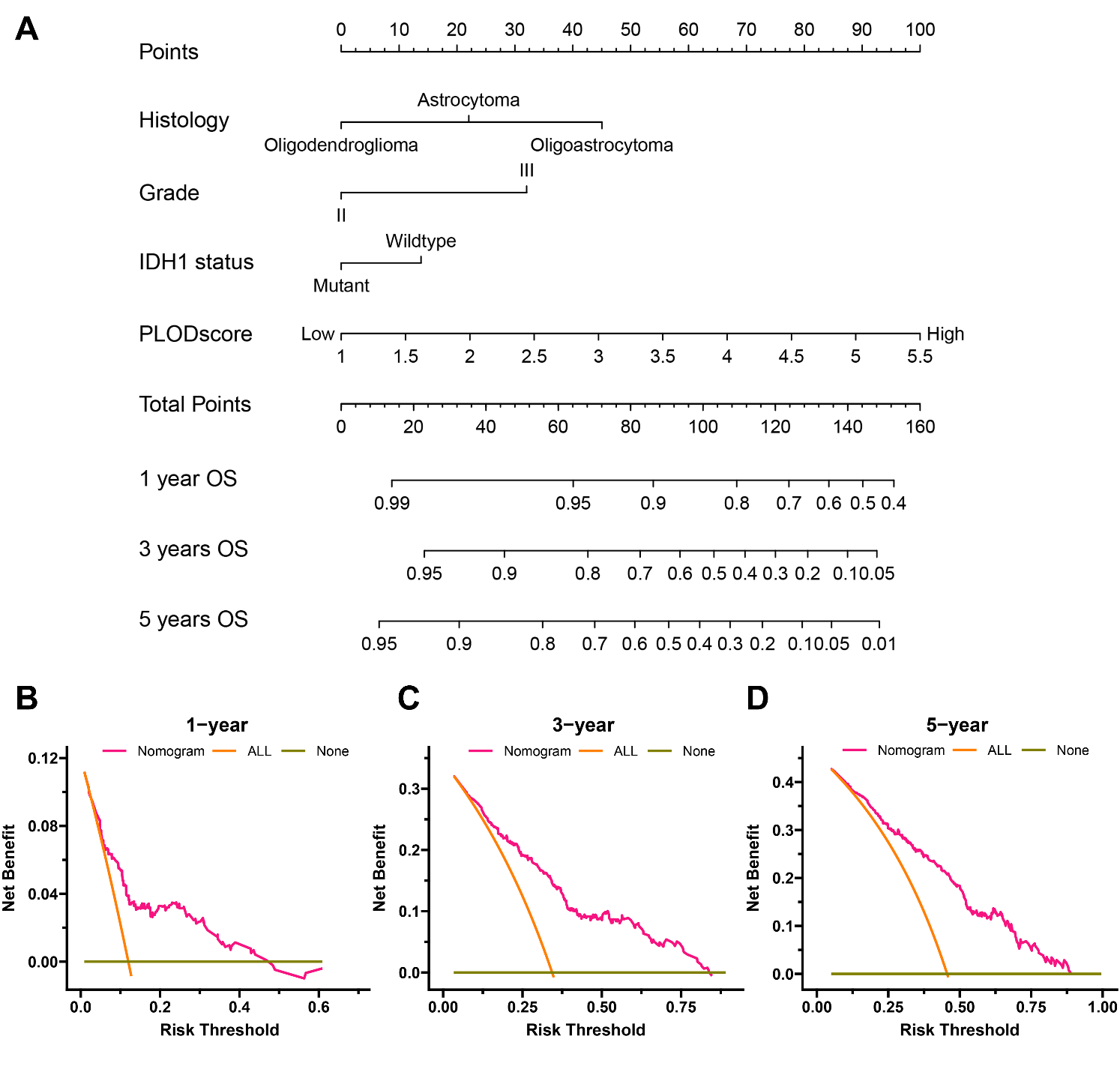


**Supplementary Figure S5. The nomogram and** **Decision Curve Analysis (DCA). (A)** Nomogram using the 4 independent factors based on multivariate analysis from CGGA. **(B)** Decision Curve Analysis (DCA) of 1-year, **(C)** 3-year and **(D)** 5-year


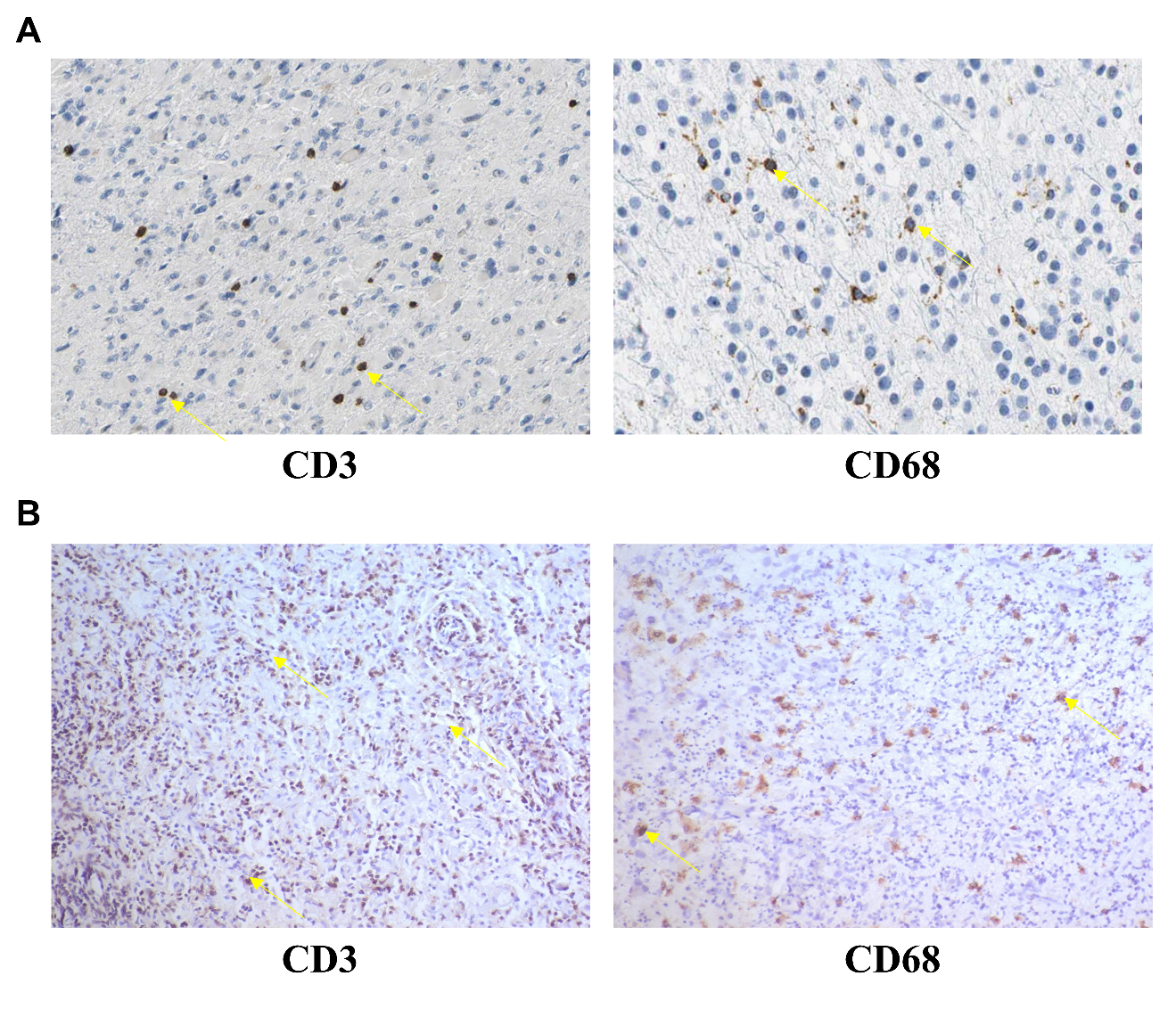


**Supplementary Figure S6. The immunohistochemical staining of CD3 and CD68 in glioma tissues. (A)** CD3 and CD68 staining from HPA dataset. **(B)** CD3 and CD68 staining using patient-derived tissues.


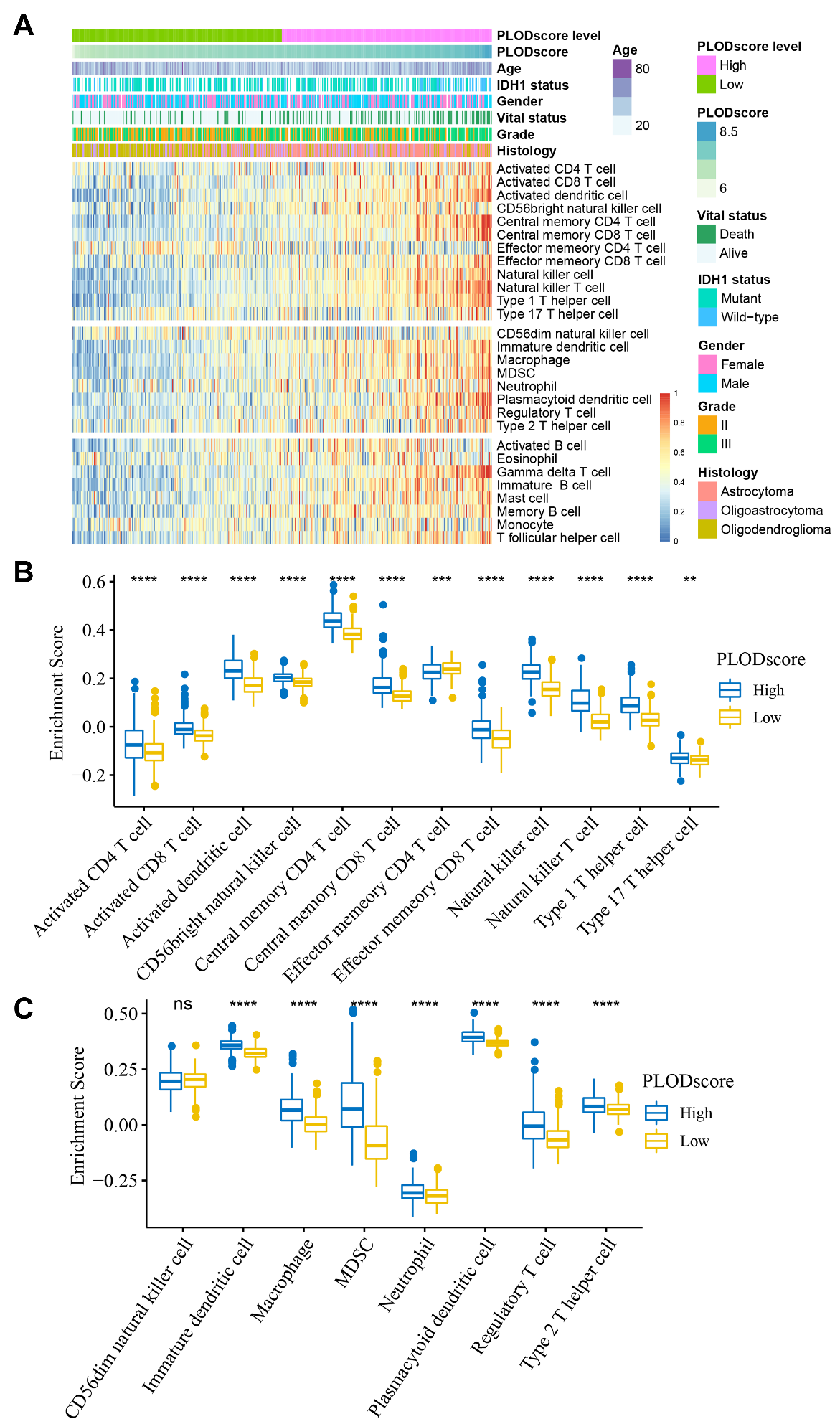


**Supplementary Figure S7.** Cellular characteristic of PLODscore based on TCGA cohort. **(A)** Multivariate statistics for PLODscore including vital status, IDH1 status, Gender, Gender, Grade of LGG, Histology of LGG and the heatmap of 28 previously reported immune cell signatures scores. **(B)** The relation between PLODscore and enrichment score of immunostimulatory cells. **(C)** The relation between PLODscore and enrichment score of immunosuppressive cells. **: *p*<0.01, ***: *p*<0.001, ****: *p*<0.0001, ns: not significant


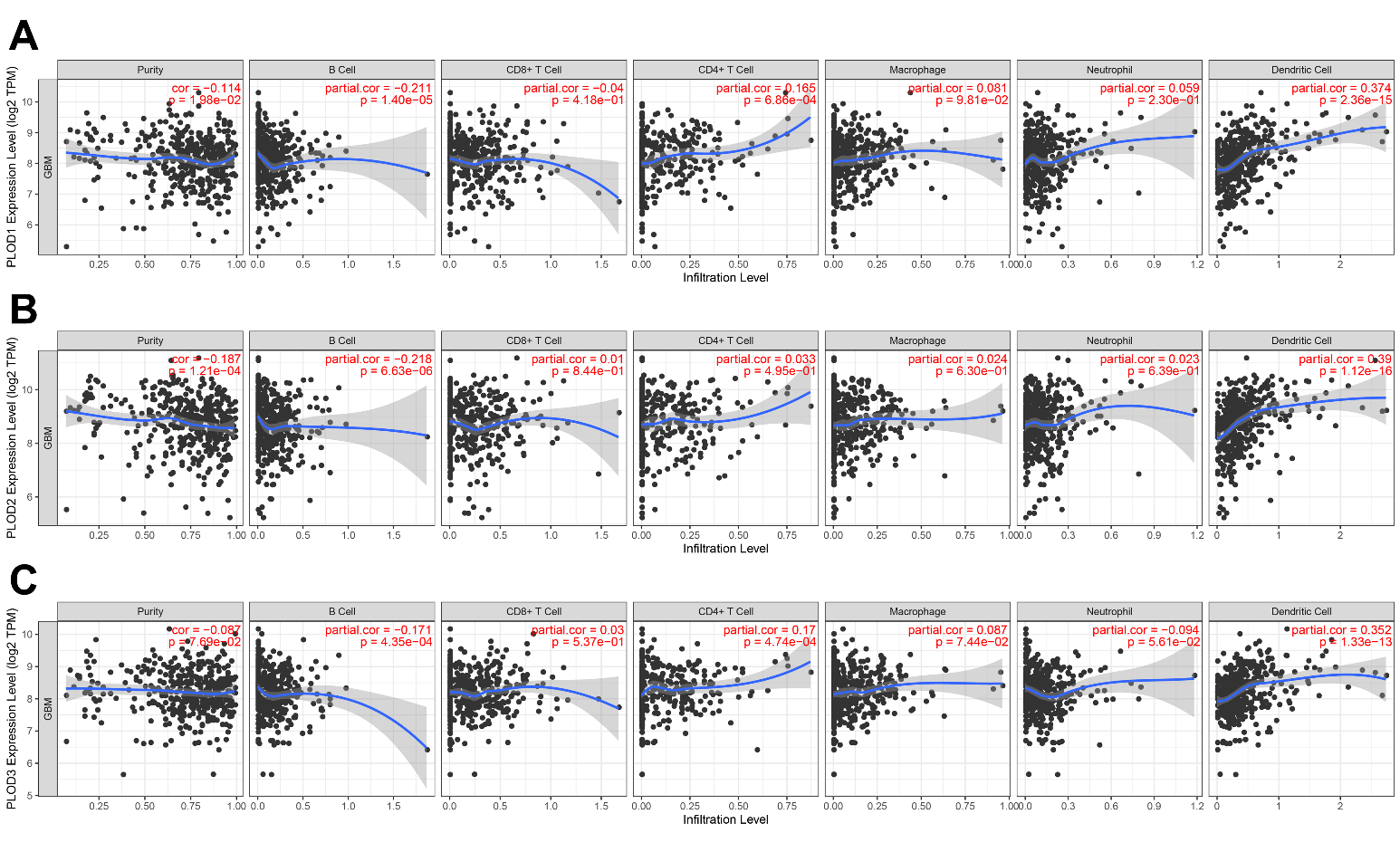


**Supplementary Figure S8.** **Correlation of PLODs expression with infiltration of six immune cell types. (A-C)** Correlation of PLOD1 (**A**), PLOD2 (**B**) and PLOD3 (**C**) expression levels with infiltration of six immune cells


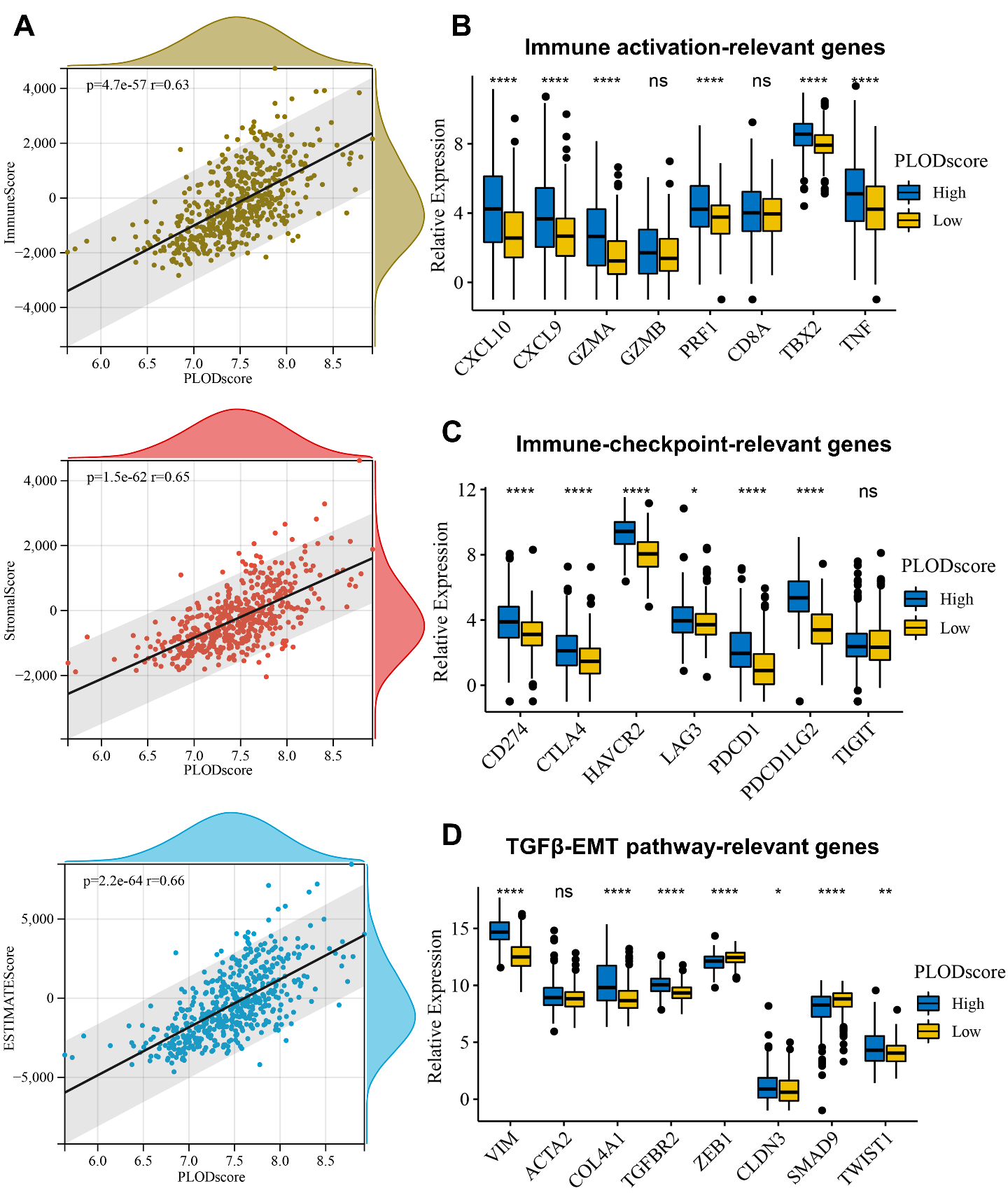


**Supplementary Figure S9.** The overview of PLODscore with immune infiltration according to TCGA database. **(A)** The association of PLODscore with immune score, stromal score and Estimate score. **(B)** The relation of PLODscore to immune activation-relevant genes, **(C)** immune-checkpoint-relevant genes and **(D)** TGF-β/EMT pathway-relevant genes. *: *p*<0.05, **: *p*<0.01, ****: *p*<0.0001, ns: not significant

## Supplementary Tables

**Supplementary table S1.** Summary of clinical characteristics for patients included in this study.

|  | **TCGA cohort (LGG, N=510)** | **CGGA cohort (LGG, N=431)** |
| --- | --- | --- |
| **Age (%)** |  |  |
| ≤40 years | 252 (49) | 220 (51) |
| >40 years | 260 (51) | 211 (49) |
| **Gender (%)** |  |  |
| Female | 205 (40) | 189 (44) |
| Male | 265 (52) | 242 (56) |
| NA | 40 (8) | - |
| **Grade (%)** |  |  |
| II | 226 (44) | 180 (42) |
| III | 244 (48) | 251 (58) |
| NA | 40 (8) | - |
| **Histology (%)** |  |  |
| Astrocytoma | 193 (38) | 263 (61) |
| Oligoastrocytoma | 129 (25) | 29 (7) |
| Oligodendroglioma | 188 (37) | 139 (32) |
| **Vital status (%)** |  |  |
| Alive | 389 (76) | 242 (56) |
| Dead | 121 (24) | 189 (44) |
| **IDH1 status (%)** |  |  |
| Mutant | 218 (43) | 297 (69) |
| Wild-type | 68 (13) | 96 (22) |
| NA | 224 (44) | 38 (9) |
|  |  |  |

**Supplementary table S3.** C-index of clinical parameters in this study.

|  | **TCGA cohort** | **CGGA cohort** |
| --- | --- | --- |
| **Age** | 0.652 | 0.517 |
| **Gender** | 0.506 | 0.510 |
| **Grade** | 0.654 | 0.610 |
| **Histology** | 0.595 | 0.631 |
| **IDH1 status** | 0.621 | 0.600 |
| **PLODscore** | 0.708 | 0.676 |

**Supplementary table S4.** The IHC staining of CD3 and CD68 in 9 glioma samples.

| Patient # | CD3 | CD68 |
| --- | --- | --- |
| #7 | Moderate | Low |
| #8 | Moderate | Low |
| #10 | High | Moderate |
| #11 | Low | Low |
| #13 | Low | Low |
| #16 | High | Moderate |
| #27 | Moderate | High |
| #28 | Low | Moderate |
| #29 | Moderate | Low |
